# Stable and Specific Multiprotein Complexes with High Molecular Masses in Different Organs of the Sea Cucumber *Eupentacta fraudatrix*

**DOI:** 10.3390/ijms27146288

**Published:** 2026-07-15

**Authors:** Svetlana E. Soboleva, Pavel S. Dmitrenok, Georgy A. Nevinsky

**Affiliations:** 1 Institute of Chemical Biology and Fundamental Medicine, Siberian Division of Russian Academy of Sciences, Lavrentiev Ave. 8, Novosibirsk 630090, Russia; 2 G. B. Elyakov Pacific Institute of Bioorganic Chemistry, Far Eastern Branch of the Russian Academy of Sciences, 159 Pr. 100 let Vladivostoku, Vladivostok 690022, Russia; paveldmt@piboc.dvo.ru

**Keywords:** sea cucumbers *Eupentacta fraudatrix*, very stable multiprotein complexes from different organs, proteins and peptides of the complexes

## Abstract

We have found for the first time several distinct, very large (1.25–1.69 megadaltons), stable protein–peptide complexes in various organs and tissues (upper body wall, gonads, respiratory trees, gut, and coelomic fluid) of the sea cucumber *Eupentacta fraudatrix*. It has been previously shown that complexes isolated from the whole organism are destroyed only under very harsh conditions. Here, for the first time, complexes were isolated from individual organs of the sea cucumber *Eupentacta fraudatrix*. According to gel filtration data, the molecular weights (MWs) of complexes from different organs are distinct but comparable (in kDa): 1690 ± 29 (coelomic fluid), 1650 ± 307 (gut), 1550 ± 253 (gonads), 1520 ± 140 (body wall), and 1250 ± 97 (respiratory trees). All complexes contain many of the same as well as different proteins with MWs of 10–20 kDa and >20 kDa. The number and type of proteins in the 10–20 kDa range vary among organs: body wall (20), gonads (19), coelomic fluid (16), gut (13), and respiratory trees (14). In addition, each complex contains specific proteins and peptides that are not present in complexes from other organs, and the number of such unique proteins also differs: body wall (13), gonads (7), respiratory trees (5), gut (4), and coelomic fluid (0). In the entire set of complexes isolated from the whole organism, 469 oligopeptides were identified, whereas only 370 peptides were detected in complexes from the five analyzed individual organs. The number of various peptides with MWs below 10 kDa in individual organ complexes decreases in the following order: body wall (134) > gut (115) > coelomic fluid (95) > respiratory trees (88) > gonads (70). These complexes contain both shared and distinct oligopeptides. Such highly stable complexes from different organs of sea cucumber *Eupentacta fraudatrix* have been isolated and characterized for the first time. In contrast to individual proteins and peptides, multiprotein complexes have expanded functional possibilities, as they can interact with various molecules and cells with which the individual proteins, enzymes, and peptides comprising the complexes can interact. Thus, they can perform the functions of all components located on their surfaces. One may suppose that many of the specific proteins, peptides, and enzymes within each complex are necessary for carrying out the specific biological functions of each individual organ.

## 1. Introduction

Vertebrates are characterized by a limited capacity for regeneration, being able to restore only certain organs (e.g., epidermis, fins, cornea, and liver) [1]. In contrast, invertebrates can regenerate various body fragments and even the entire body [2,3,4] and possess highly specific gene regulation pathways that are of great interest for regenerative medicine and biology [3]. Echinoderms, including holothurians (sea cucumbers), represent highly valuable models for investigating regeneration mechanisms and for the discovery of new therapeutic agents to restore diseased organs. Sea cucumbers exhibit a unique regenerative potential [2,3,4,5]. Therefore, the assay of biomedical, biochemical, and molecular mechanisms underlying regeneration in holothurians is of fundamental importance, particularly in light of potential applications for human regenerative medicine.

It has been established that many diverse biological processes are carried out by various protein complexes [6,7]. For instance, certain biological processes require highly specific proteins, peptides, and enzymes that associate to form larger stable or transient multiprotein complexes. Such complexes demonstrate greatly enhanced and expanded biological functions, including accelerating metabolic pathways and increasing the efficiency and specificity of action [6,7].

One of the best-known stable complexes is the ribonucleoprotein complex—the ribosome—which performs protein synthesis [8]. Interestingly, ribosomes can form numerous additional complexes with different proteins and nucleic acids, acquiring biological functions distinct from those of native ribosomes [9,10]. Other complexes involved in signal transduction [11], cellular transcription and post-transcriptional modifications [12], and translational control have also been described [13]. For example, the brain-derived neurotrophic factor (BDNF) complex is an important regulator of synaptic transmission in various brain regions, including the hippocampus [14].

Several other stable protein complexes have also been described. The Elg1 replication factor C-like multicomplex is essential for genome stability [15]. A special multi-subunit protein complex plays a fundamental role in eukaryotic mRNA metabolism and has a multitude of functions that impact eukaryotic gene expression [16]. Stable Polycomb multiprotein complexes are very important as chromatin regulators in eukaryotic gene transcription [17]. Several stable complexes important for quality-control pathways of protein import into mitochondria have also been described [18]. Several specific complexes are also important for the functioning of signaling and regulatory proteins [19]. A multiprotein complex of the respiratory chain has been described [20]. Human membrane-associated complexes from the placenta, containing different proteins, were analyzed by SDS-PAGE and MALDI mass spectrometry; 34 novel heterooligomeric protein complexes were identified [21].

Authors of [22,23,24,25,26,27,28,29,30,31,32] discuss the basic physical–chemical principles underlying the formation of stable and unstable macromolecular complexes.

Numerous bioactive compounds are present in the human placenta, milk, and other organs of various organisms. A key question is which peptides, proteins, and other molecules from different cells, biological fluids, and organs can form temporary or even stable associations with distinct biological functions. Recently, highly stable complexes with high molecular weights (MWs) have been discovered for the first time. Specifically, very stable multiprotein complexes (SPCs; approximately 1000 ± 100 kDa) were identified in human milk [33], placentas [34,35], sea urchin eggs [36], and the whole organism of the sea cucumber *Eupentacta fraudatrix* [37]. These complexes contain various proteins with low, moderate, and high molecular masses, as well as numerous peptides with MWs below 10 kDa [33,34,35,36,37]. Notably, despite both being derived from female organisms, the multiprotein complexes of human milk [33] and placentas [34,35] differ greatly in their protein and peptide composition.

All complexes are very stable in the presence of 1.0–3.0 M NaCl and 2–4 M urea but dissociate in the presence of 8.0 M urea supplemented with 2.0–3.0 M NaCl, EDTA, and DTT [33,34,35,36,37]. The formation of such very stable complexes cannot be the result of a random association of proteins.

We hypothesized that the recently discovered, very new type of extremely stable protein complexes may also exist in different biological fluids of various organisms. It is particularly important that the formation of stable multiprotein complexes could lead to a vast expansion of their biological properties and functions, including their associations with different cells, proteins, peptides, nucleic acids, oligosaccharides, and other molecules. Therefore, the search and investigation of undescribed stable complexes with extended biological functions are of particular interest.

Previously, we had obtained and studied very stable complexes from human placentas, milk, sea urchin eggs, and the whole organism of the sea cucumber *Eupentacta fraudatrix* [33,34,35,36,37]. However, it was of interest to determine whether such complexes can exist in different organs of various organisms and, if so, whether they differ in composition and other features. With this in mind, we recently isolated for the first time highly stable complexes from various organs (body wall, gonads, respiratory trees, gut, and coelomic fluid) of the sea cucumber *Paracaudina chilensis* [38]. The results were somewhat unexpected. All stable complexes contained many proteins (>10 kDa), the number and composition of which varied among organ complexes. Additionally, the whole-organism complexes from *P. chilensis* contained 254 different peptides (<10 kDa). The peptide content in organ-specific stable complexes increased in the following order: gonads (55) < gut (58) < body wall (64) < coelomic fluid (76) < respiratory trees (104). These data indicated that, in other organisms as well, recently discovered highly stable complexes may differ significantly among different organs.

In this work, therefore, we isolated multiprotein complexes not only from the whole organism of the sea cucumber *Eupentacta fraudatrix* but also from individual organs and tissues (body wall, respiratory trees, coelomic fluid, gonads, and gut). New and interesting results were obtained. It was shown that all these organs contain very stable multiprotein complexes, but depending on the organ, they differ in their protein and oligopeptide (OP) composition. This represents the second example demonstrating that different organs of the same organism can contain various stable complexes of proteins and peptides.

## 2. Results

### 2.1. Isolation of Protein Complexes from Different Organs

In a previous study [37], a protein complex was obtained from the homogenate of the whole organism of the sea cucumber *Eupentacta fraudatrix* by gel filtration on Sepharose 4B, which productively separates proteins with molecular weights (MWs) in the range of 60–20,000 kDa. The whole-body (containing all organs) complex was shown to have a MW of approximately 2.0 MDa (Supplementary Figure S1) and to be very stable (Supplementary Figure S2). It is effectively dissociated only in a mixture of 8.0 M urea or 3.0 M MgCl_2_ containing EDTA and DTT. Recently, we isolated and characterized for the first time highly stable protein complexes from the whole organism and various organs of the sea cucumber *Paracaudina chilensis* [38]. All complexes from different organs differed significantly in protein and peptide composition. In addition, protein complexes from the maternal placenta and milk also consist of different proteins and peptides [33,34,35]. It was therefore of interest to determine whether individual organs of different organisms, including the sea cucumber *E. fraudatrix*, contain the same or different multiprotein complexes.

**Figure 1:**
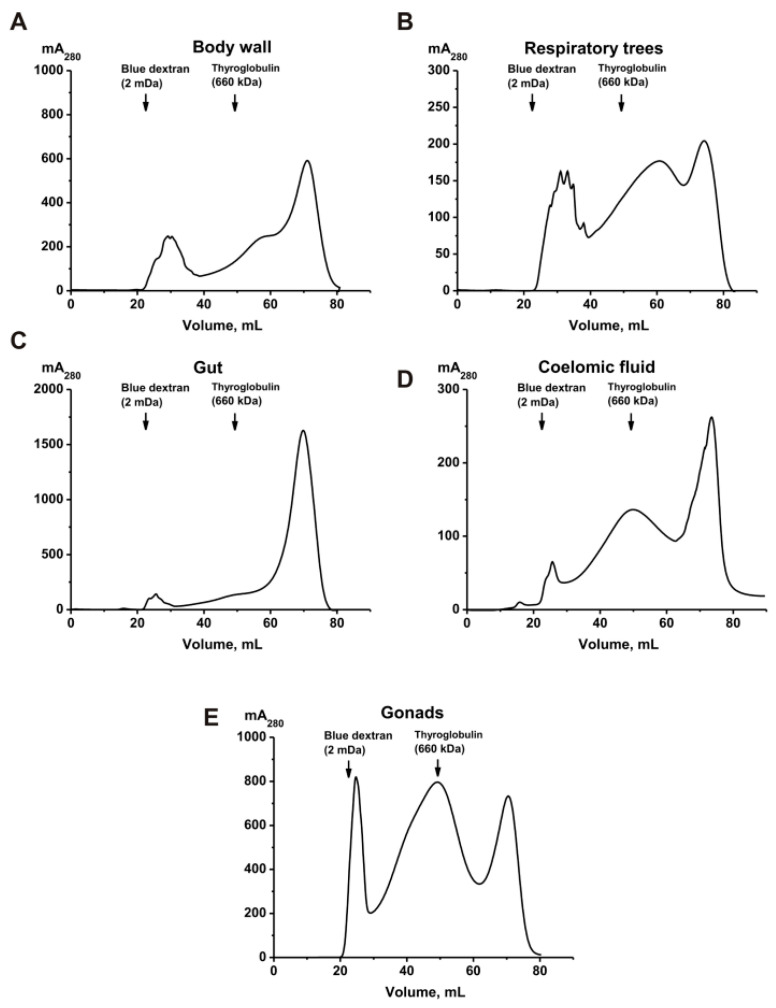
Isolation of several different multiprotein complexes from various organs of the sea cucumber *E. fraudatrix* by FPLC gel filtration on a Sepharose 4B column using homogenates of different organs: body wall (**A**), respiratory trees (**B**), gut (**C**), coelomic fluid (**D**), and gonads (**E**).

In the present study, intact sea cucumbers *E. fraudatrix* and mixtures of coelomic fluid and different body parts—namely (a) body wall, (b) respiratory trees, (c) gut, (d) coelomic fluid, and (e) gonads—were used. Mixtures of 5–7 intact organisms and mixtures of isolated samples of different organs and tissues from 35–40 sea cucumbers were subjected to homogenization as described in [37,38]. All homogenates were then subjected to FPLC gel filtration on Sepharose 4B. Representative gel filtration profiles are presented in Figure 1.

All homogenates from different organs were subjected to FPLC gel filtration under identical conditions. Supplementary Figure S1 demonstrates data on the gel filtration of a preparation corresponding to the whole body of the sea cucumber. In all cases, the first peaks correspond to high-molecular-weight protein complexes (Figure 1). Overall, all gel filtration profiles are similar to some extent and differ only in the relative quantity and number of peaks of protein material in the second and third peaks. However, the average MWs of the complexes corresponding to the first peak after gel filtration were slightly different; that of the whole organism with all organs was approximately 2.0 ± 0.4 MDa [37]. Molecular weights of complexes from different organs according to gel filtration data are to some extent comparable (in kDa): 1690 ± 29 (coelomic fluid), 1650 ± 307 (gut), 1550 ± 253 (gonads), 1520 ± 140 (body wall), and 1250 ± 97 (respiratory trees). However, the close MW values of some complexes did not indicate identical composition of these complexes.

### 2.2. SDS-PAGE Assay of Complex Proteins

**Figure 2:**
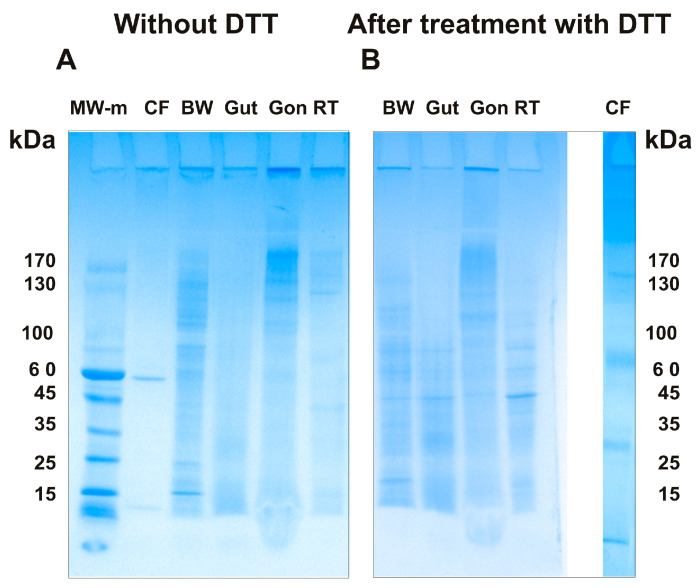
SDS-PAGE investigation of proteins (9–18 µg) from sea cucumber multiprotein complexes of various organs using a 4–18% gradient gel before (**A**) and after (**B**) treatment of all complexes with DTT: BW—body wall; CF—coelomic fluid; and RT—respiratory trees. After treatment with DTT, the complex from coelomic fluid was analyzed using additional gel. Molecular weight markers are indicated on the figure. See Section 4—Materials and Methods for additional details.

Analysis of proteins in very stable complexes is a challenging task. This difficulty arises because even SDS does not completely dissociate such highly stable complexes, and some high-molecular-weight associates may remain in the wells of the gels during SDS-PAGE. Proteins of the complexes corresponding to different organs were first analyzed using SDS-PAGE (Figure 2).

The summarized data from the assay of major proteins in the sea cucumber complexes using SDS-PAGE before and after treatment of these complexes with DTT (three independent experiments) are presented in Table 1.

Complexes from different organs have distinct but comparable MWs. At the same time, they differ partially or significantly in the composition of protein bands before and after treatment of the complexes with DTT. A total of 3 to 12 major proteins, corresponding to individual proteins or their associates, were found in the five proteins of the complexes before treatment with DTT. Several major protein bands with high MWs (280, 240, or 215 kDa) were detected in the complexes prior to DTT treatment. However, these bands disappeared after treatment of the complexes with DTT, and smaller proteins appeared. This indicates that some of the protein bands observed before DTT treatment may correspond to small protein associates in which proteins are linked by disulfide (S–S) bonds.

After treatment of the complexes with DTT, the greatest number of major proteins (10) was found in the complex from the body wall and the smallest number (5) in the complex from the gonads (Table 1). Three major proteins (approximately 15, 44, and 68 kDa) were revealed in all complexes after DTT treatment, with the exception of the coelomic fluid. Conversely, the protein with MW of approximately 60 kDa was found in all complexes except that from the gonads. Some complexes contain individual major proteins not detected in any other complex: coelomic fluid (140 ± 7.4, 40 ± 2.9, 36 ± 2.0, and 10 ± 1.2 kDa), body wall (131 ± 6.5 and 70 ± 2.8 kDa), gonads (122 ± 6.1 kDa), and respiratory trees (59 ± 3.5 kDa). Overall, all complexes from different organs, both before and after DTT treatment, differ significantly (Table 1). However, SDS-PAGE allows determination of only the major proteins present in the stable complexes. Therefore, we performed an analysis of proteins in the stable complexes from different organs using MALDI mass spectrometry.

### 2.3. MALDI Mass Analysis of Proteins

**Figure 3:**
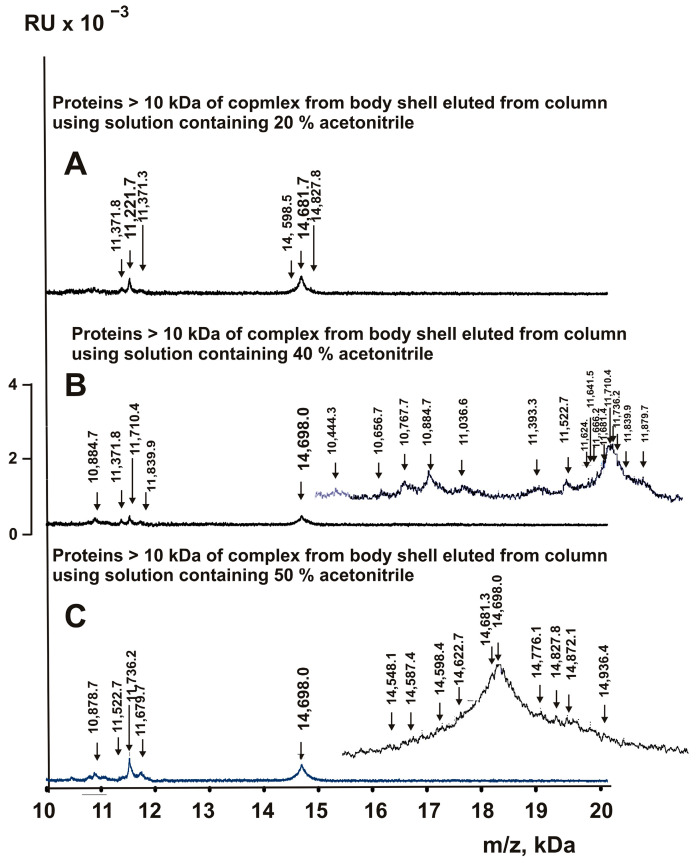
MALDI mass spectra of 10–20 kDa proteins from the body wall complex eluted from C18 tips using different concentrations of acetonitrile: 20% (**A**), 40% (**B**), and 50% (**C**). The inset in panel (**B**) shows a more detailed spectrum corresponding to the 10–12 kDa region, and the inset in panel (**C**) corresponds to the 14–15 kDa region.

SDS-PAGE analysis does not permit effective separation of proteins with nearly identical or comparable MWs. Therefore, we estimated the MWs of proteins in 10–20 kDa range by MALDI mass spectrometry. Analysis of the complex mixtures of various proteins by this method is very challenging. If any component effectively crystallizes with the matrix, it suppresses the formation of crystals with the other components and hinders the detection of well-defined peaks corresponding to other proteins and peptides. When intact complexes were used directly for analysis, only peaks corresponding to some major proteins were reliably detected in the spectra, and the spectra were poorly resolved. Consequently, each of the complexes from various organs was first applied to ZipTip Pipette Tips C18 for reversed-phase chromatography. The proteins and peptides of the complexes were eluted from the sorbent sequentially with solutions containing 5.0, 10.0, 20.0, 30.0, 40.0, 50.0, 70.0, 80.0, and 90.0% acetonitrile. The eluted fractions from all stable protein complexes were analyzed for proteins by MALDI mass spectrometry using a special mode for the analysis of proteins with MWs of 10–20 kDa. For each of the nine fractions eluted from sorbent, 8–10 spectra were obtained. Figure 3 shows, as an example, the spectra of several fractions corresponding to the complex from the body wall.

**Figure 4:**
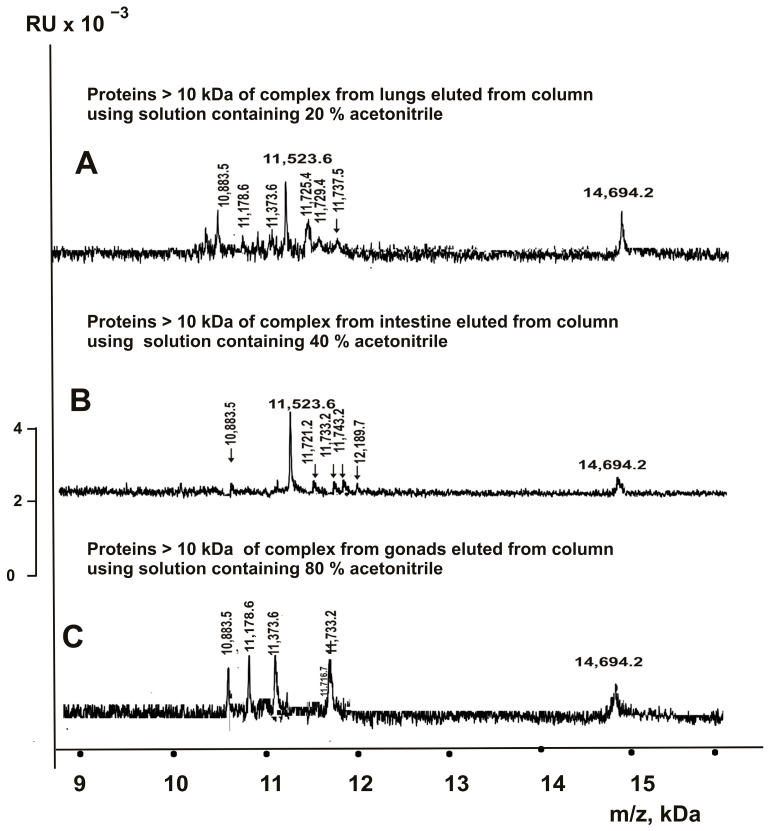
MALDI mass spectra of 10–20 kDa proteins from complexes of three different organs eluted from C18 tips using different concentrations of acetonitrile: (**A**) respiratory trees (20%), (**B**) gut (40%), and (**C**) gonads (80%).

It should be noted that presenting spectra using molecular mass ranges from 10 to 20 kDa does not allow visualization of all protein peaks with closely spaced values. Therefore, more detailed spectra of selected peaks are additionally shown in the insets of panels B and C. Spectra of this type (Figure 3) were obtained for each of the fractions eluted with different concentrations of acetonitrile from C18 tips for complexes from all organs. Representative examples are shown in Figure 4.

Based on the analysis of all 8–10 spectra obtained for each complex, corresponding to fractions eluted from columns with 5–90% acetonitrile for every organ, the MWs of the proteins were estimated (Table 2).

In the set of proteins with MWs >10 kDa from all complexes of the whole organism, 50 proteins were detected using MALDI mass spectrometry (Table 2). The maximum number of proteins (20) was detected in the complex from the body wall, and the number decreases in the following order: body wall (20) > gonads (19) > coelomic fluid (16) > respiratory trees (14) > gut (13).

One major protein with MW = 15,876 Da was found in all five complexes from the individual organs. Interestingly, according to SDS-PAGE data, various complexes contain proteins with MWs ranging from 15 ± 0.8 to 16 ± 0.9 kDa (Table 1). It is reasonable to expect that these proteins correspond to those with MWs of 15,132.2 ± 2.4, 15,738.8 ± 2.5, and 15,876.5 ± 2.1 Da, as revealed by MALDI mass spectrometry (Table 2). Unexpectedly, a protein with MW of 14.5–14.8 kDa was not detected in the complexes by SDS-PAGE; however, 18 proteins with MWs ranging from 14,532.5 ± 2.3 to 14,765.5 ± 2.2 Da were detected using MALDI mass spectrometry (Table 2). The protein with MW of 10.0 ± 3.2 kDa was reliably detected by SDS-PAGE only in the complex from coelomic fluid. Nevertheless, at least one of the eight proteins with MWs in the range of 10.7–10.9 kDa was detected by MALDI mass spectrometry in all five complexes (Table 2).

It should be noted that some proteins have very close MWs. However, Table 2 includes only those proteins that correspond to closely spaced peaks within the same spectrum. Furthermore, proteins were included in Table 2 only if they were detected in 8–10 spectra corresponding to the same eluate from the reversed-phase column. Nevertheless, it cannot be ruled out that some proteins with comparable MWs are the same major or intermediate proteins with minor modifications, such as glycosylated, phosphorylated, or acylated forms.

Some complexes from different organs contain proteins that are also found in one or more other organ complexes. However, each complex contains unique proteins specific to that organ, and the number of such organ-specific proteins differs noticeably: body wall (13), gonads (7), respiratory trees (5), gut (4), and coelomic fluid (0) (Table 2).

Thus, it is evident that proteins with MWs of 10–20 kDa may be the same or different across the various complexes from different organs, but a major protein with MW = 15.1–15.9 kDa is common to all complexes. It cannot be excluded that these proteins play an important role in the formation of stable complexes in all organs.

### 2.4. Peptides of the Stable Complexes

As demonstrated previously, stable protein complexes from sea urchin eggs, human milk, placenta, and the sea cucumber *Paracaudina chilensis* contain, in addition to proteins (>10 kDa), numerous peptides with MWs below 10 kDa [33,34,35,36,37,38]. Earlier, it was shown that the stable complex from the whole organism of *Eupentacta fraudatrix* also contains proteins (>10 kDa) and various peptides (<10 kDa) [37]. In the present study, a more detailed analysis of peptides in complexes from different organs of *E. fraudatrix* was carried out for the first time.

As noted above, analysis of the MWs of complex mixtures containing many different proteins and peptides is a very challenging task. However, oligopeptides (OPs) with MWs below 10 kDa can sometimes be reliably detected even in relatively complex mixtures using an α-cyano-4-hydroxycinnamic acid matrix [39]. Nevertheless, this is highly dependent on the specific peptides present in the analyzed mixtures. Some peptides that crystallize well with the matrix used may suppress the detection of other OPs.

**Figure 5:**
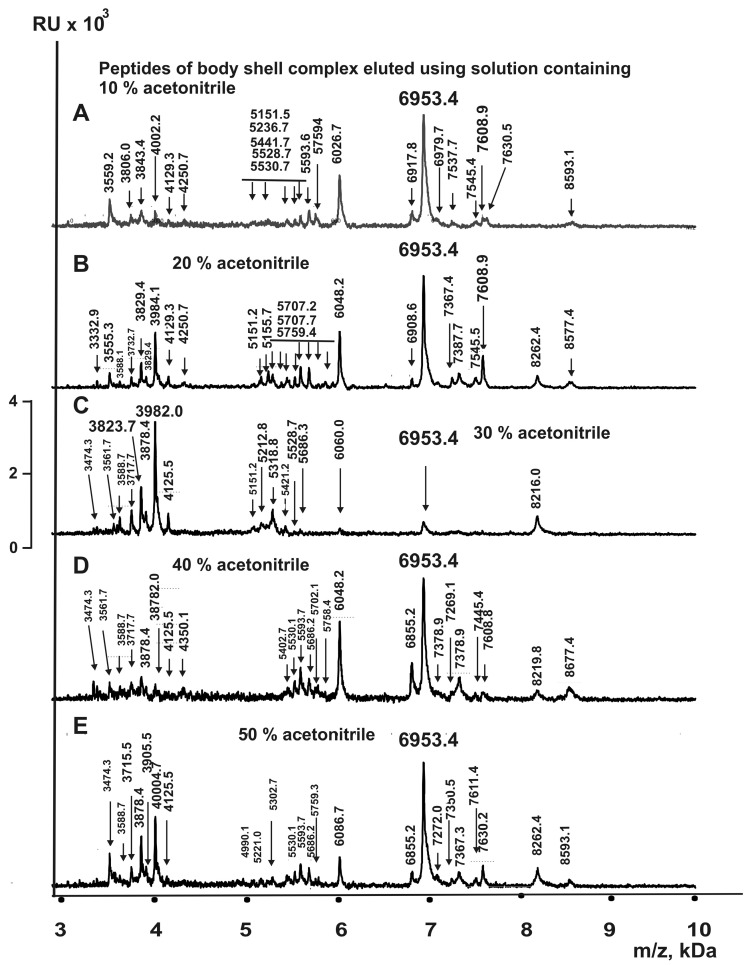
MALDI mass spectra of oligopeptides (OPs) in the 3–10 kDa range, eluted from a C18 tip column from the complex of body wall, using solutions containing different concentrations of acetonitrile: (**A**) 10%, (**B**) 20%, (**C**) 30%, (**D**) 40%, and (**E**) 50%.

First, we analyzed the peptides in the complex from the whole organism and from the five organs by applying aliquots of all complex solutions to the ion targets for MALDI mass analysis. The spectra obtained contained peaks corresponding to some oligopeptides with MWs below 10 kDa, but they were very poorly resolved. Therefore, for a more detailed analysis of OPs, we used thin-layer chromatography with ZipTip C18 tips (as described above). Proteins and OPs were eluted from the sorbent with solutions containing 5, 10, 20, 30, 40, 50, 70, 80, and 90% acetonitrile. Nine fractions from the stable complex of the whole organism and from the complexes of the five organs were analyzed for peptide detection by MALDI mass spectrometry using a special mode for OPs in the 2–10 kDa range. For each fraction corresponding to the nine eluates, 8–10 independent spectra were analyzed. As an example, Figure 5 shows several spectra corresponding to five eluates from the stable complex of the body wall.

**Figure 6:**
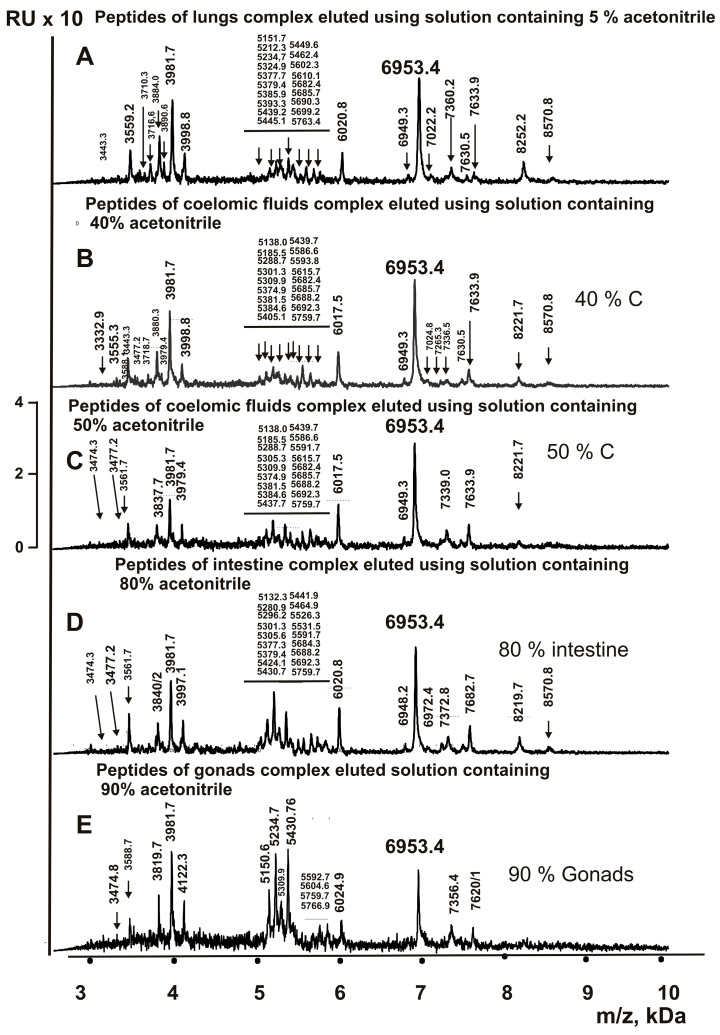
MALDI mass spectra of 2.5–10 kDa OPs eluted from a C18 tip column using solutions containing different concentrations of acetonitrile for complexes from respiratory trees ((**A**) 5%), coelomic fluid ((**B**) 40%), coelomic fluid ((**C**) 50%), gut ((**D**) 80%), and gonads ((**E**) 90%). In some regions, numerous peaks with closely spaced MWs were observed; the *m*/*z* values of these peptides are indicated above the lines.

MALDI mass spectra of OPs in the 3–10 kDa range eluted from the sorbent with solutions containing 5–90% acetonitrile were obtained for the complexes from all organs analyzed. As an example, Figure 6 shows representative spectra corresponding to different organs and different concentrations of acetonitrile.

The MWs of peptides in fractions eluted from columns with 5–90% acetonitrile were estimated. When MWs of OPs were comparable, they were considered reliable only if they corresponded to very closely spaced peaks within the same spectrum. The average MW for each peptide was estimated using 8–10 spectra for each of the fractions eluted with 5–90% acetonitrile. The fractions corresponding to the stable complex from the whole organism (containing complexes from all organs of the sea cucumber) contained 469 oligopeptides with MWs ranging from 3.0 to 8.9 kDa (Supplementary Table S1). Complexes from individual organs analyzed in this study contained a smaller number of oligopeptides, decreasing in the following order: body wall (134) > gut (115) > coelomic fluid (95) > respiratory trees (88) > gonads (70) (Supplementary Table S2). Only four of the same major peptides were found in complexes from all five organs, with MWs of 6953.4, 5151.7, 4129.8, and 3474.3 Da. Four oligopeptides (5759.7, 3983.6, 3981.7, and 3825.5 Da) were common to four complexes (Supplementary Table S2). Some oligopeptides were detected in three or two complexes. Furthermore, all five complexes contained several individual OPs that were not found in any other complex (Supplementary Table S2).

Thus, we obtained highly unexpected and novel data regarding the large number of different oligopeptides present within the stable complexes of various organs of the sea cucumber. Currently, there are no data on very stable multiprotein complexes from various organs of any different mammals, with the exception of the sea cucumber *Paracaudina chilensis*. It is possible that in different organisms, protein complexes may differ in composition and biological properties depending on the organ.

## 3. Discussion

Analyses were performed previously on stable protein complexes from mothers’ milk and placenta [33,34,35], sea urchin eggs [36], as well as from the whole organisms of the sea cucumbers *E. fraudatrix* [37] and *P. chilensis*, and from different organs of *P. chilensis* [38]. Determination of the components of this new type of complex from the whole organisms of *P. chilensis* and *E. fraudatrix* demonstrates a novel principal finding: all of these very stable complexes contain a large number of proteins and peptides [33,34,35,36,37,38]. Since different organs of all organisms perform distinct biological functions, it was of interest to determine whether extremely stable protein complexes exist in different organs of *E. fraudatrix* and, if so, whether they differ in protein and oligopeptide composition.

In this study, the investigation of stable protein complexes from five different organs of the sea cucumber *E. fraudatrix* was performed for the first time. It should be emphasized that, according to gel filtration results, all five stable complexes differ in their MWs only moderately (Figure 1). All protein complexes from the whole organism of *E. fraudatrix* are stable [37], similar to the stable associates from other investigated organisms [33,34,35,36,37,38]. These data indicate that hydrogen bonds, electrostatic interactions, and metal-dependent contacts are formed between the proteins and peptides of the stable complexes, similar to what has been observed for complexes of sea urchin eggs, milk, and placenta [33,34,35,36,37,38].

Data demonstrating significant differences in protein composition among different organs of holothurians were obtained by analyzing proteins using SDS-PAGE (Figure 2) and MALDI mass spectrometry (Table 1). The five complexes contain different numbers of major proteins according to SDS-PAGE analysis (Table 1).

Analysis of all complexes from the whole organism of the sea cucumber *Eupentacta fraudatrix* by MALDI mass spectrometry of eluates from the sorbent revealed 50 proteins with MWs of 10–20 kDa (Table 2). However, all five multiprotein complexes from individual organs differ in their content of various proteins. An even more unexpected finding was the discovery of 469 oligopeptides (OPs) with MWs ranging from 3 to 10 kDa in the set of stable complexes from the whole organism (Supplementary Table S1). At the same time, specific protein complexes from various organs contain different numbers of OPs (Supplementary Table S2). Only a few oligopeptides are common to complexes from all five organs. Notably, each of the five complexes contains individual organ-specific OPs (Supplementary Table S2).

Thus, stable complexes from all organs differ both in their content of proteins with MWs > 10 kDa and in OPs with MWs < 10 kDa.

As indicated above, in contrast to individual proteins, multiprotein complexes have expanded possibilities for interaction due to their ability to bind with various molecules in biological fluids and with cells. Consequently, the complexes can provide the functions of all molecules located on their surfaces. In cells and biological fluids of various organisms, there are components specific to each organ and biological fluid.

We have obtained data on several enzymatic activities of the complexes from different organs: protease, DNase, RNase, phosphatase, and amylase (to be published elsewhere). These enzymes, as components of the complexes from different organs, differ in their number, molecular weights, pH optima, and dependence on or independence from mono- and divalent metal ions.

These specific components are necessary for carrying out the specialized functions of each individual organ. Taking this into account, it can be assumed that multiprotein complexes in different biological fluids and cells may also differ accordingly.

## 4. Materials and Methods

### 4.1. Reagents

Adult *E. fraudatrix* sea cucumbers were collected from Peter the Great Bay of the Japan Sea as described in [37]. These sea cucumbers are primarily found on the seafloor, 2–10 m below the surface. The average salinity is approximately 34.5‰ (ppm) (https://oceanography-danchenkov.ru/oceanography/peter-the-great-bay-introduction/, published on 26 April 2017, accessed on 8 July 2026).

The pressure at a depth of 2–10 m varies from 68,600 to 147,000 Pascals, and the temperature is approximately 7–0 °C in summer and 5–10 °C in winter.

Coelomic fluids, body wall, gut, respiratory trees, and gonads were isolated from sea cucumbers before they were frozen. Then, all samples of sea cucumber’s organs were frozen and stored (−40 °C) until the experiments. High-purity compounds (Coomassie blue, EDTA, SDS, NaCl, Tris, glycerol, and some other reagents) were purchased from Sigma (St. Louis, MO, USA). Columns of Sepharose 4B were purchased from GE Healthcare Life Sciences (Marlborough, MA, USA).

### 4.2. Sea Cucumber Extracts Obtaining

To obtain homogenates of different organs, we used mixtures of intact complete bodies of these cucumbers and organs (upper body wall, gonad, respiratory trees, and gut) of sea cucumbers *Eupentacta fraudatrix* and mixtures of coelomic fluids (50 mL). Mixtures of 5–7 intact cucumbers (100 g) and mixtures of various organs isolated from 30–40 sea cucumbers (10–20 g) were used. All samples were carefully washed three times before lysis using buffer A (Tris-HCl buffer (10 mM, pH 8.0) supplemented with 0.1 M NaCl and several antibiotics (10^4^ Units/mL penicillin, 10^4^ Units/mL streptomycin and 25 Units/mL ampicillin) to inactivate possible bacteria on their surfaces. For lysis, mixtures of fragments of various organs were mixed with buffer A containing 1.0 mM DTT and 1.0 mM EDTA and all antibiotics mentioned above in a volume ratio of 1:10 and homogenized. All different samples sets were carefully homogenized using the same conditions. The homogenates obtained were centrifuged at 16,500× *g* for 40 min (Palo Alto, CA, USA); Avanti J-E centrifuge; Beckman Coulter). All supernatants were treated with ultrasound for the removal of lipid compounds using a QSonica Q125 sonicator (Newtown, CO, USA) for 10 min, at an amplitude of 30%, and then dialyzed against 2.1 L of 10 mM Tris-HCl (pH 8.0) at 4 °C, first 3 times for 4 h, and then overnight.

### 4.3. Complex Purification

It has been previously shown that the whole body of sea cucumber *E. fraudatrix* extract contains a very stable high MW protein complex (~2.0 ± 0.4 MDa) [37]. Therefore, similar to [37] for removing stable multiprotein complexes, the gomogenates of intact sea cucumber *E. fraudatrix* and their organs were subjected to gel filtration on Sepharose 4B, which efficiently separates various proteins with MWs of 60–20,000 kDa. The concentrated preparations of proteins (1 mL) were applied to a column with Sepharose 4B (90 mL) equilibrated in TBS (20 mM Tris HCl (pH 7.5) and 0.5 M NaCl) and all antibiotics using a chromatograph GE Healthcare Akta Purifier (Chicago, IL, USA). The fractions (4.0 mL) eluted from the column using the same buffer were collected. The complexes and other proteins were monitored by A_280_ (absorbance at 280 nm). Isolation of stable protein complexes from different organs (body wall, respiratory trees, coelomic fluid, gut, and gonad) was carried out using the same conditions described above for the whole organism [37]. For removing NaCl from eluates, all samples were dialyzed using 10 mM Tris-HCl, pH 7.5 for 16 h at 4 °C and then concentrated on a rotary evaporator (Техас, USA; Refrigerated Centri Vap Concentrator, Labconco, Kansas City, MO, USA). To search for the protein complexes, all preparations corresponding to the first peak after gel filtration were used. Ultracentrifugation at 100,000× *g* for 2.0 h was used to purify the preparations from admixtures of components of the second peaks (Optima XE-90 Ultracentrifuge; Beckman Coulter, Brea, CA, USA). Then all samples were used for subsequent experiments of various types. All experiments were performed under sterile conditions.

### 4.4. SDS-PAGE Assay

Analysis of protein complexes by SDS-PAGE was performed using a 5–18% gradient gel containing 0.1% SDS [37,38], according to the Laemmli method. Before electrophoresis of protein complexes, all samples (10–20 μg) were preincubated with buffer (50 mM Tris-HCl (pH 6.8), 1.0% bromophenol blue, 10 mM EDTA, 10% glycerol, 0.1% SDS) for 8 min at 100 °C and then applied to the gel. Proteins were stained using Coomassie R-250. The MWs of major proteins after staining were calculated using the data of the calibration curve corresponding to proteins with known MWs. All molecular weights (kDa) of proteins are given as the mean ± standard deviation from two to three independent SDS-PAGE (Table 1).

### 4.5. MALDI Mass Spectrometry Analysis of Proteins and Peptides

The analysis of peptides and proteins of coelomic fluids and homogenates of different organs was performed using the Reflex III system (Bruker Company; Frankfurt, Germany): 337-nm nitrogen laser VSL-337 ND with 3 ns pulse duration.

Coelomic fluid or homogenates of various organs of sea cucumber were first directly applied for MALDI mass spectrometry to an ion target as in [37,38]. The spectra of proteins and peptides were very poorly resolved, because the solutions and the protein complexes themselves contained metal ions and many other components that reduced the quality of the spectra. To improve the quality MALDI mass spectra, the solutions of all six complexes (10.0 μL) were used according to a standard procedure for reverse phase chromatography using ZIPTip Pipette Tips C18 (Sigma-Aldrich; Merck KGaA, Darmstadt, Germany). Different salts were removed from the columns by washing with 2 mL of water. Then the proteins and peptides were sequentially eluted from C18-sorbent with several solutions (200 µL) containing acetonitrile in different concentrations: 5, 10, 20, 30, 40, 50, 70, 80, and 90%. To remove acetonitrile the obtained solutions were dried. The resulting preparations of proteins and peptides were dissolved in 10 µL of water. Aliquots of the solutions (1–2 µL) obtained were used for peptide and protein analysis using MALDI mass spectrometry as described below. All MWs (Da) of different proteins are given as the mean ± standard deviation from 7–10 to three independent MALDI mass specters (Table 2).

For the analysis of oligopeptides <10 kDa and proteins >10 kDa, 1–2 μL of mixtures eluted from sorbent by acetonitrile in different concentrations were mixed with 1–2 μL of matrix dissolved in 0.1% acetonitrile and trifluoroacetic acid (1:2). For the analysis of proteins (>10 kDa) the solutions (1–2 µL) were mixed with a saturated solution of α-sinapinic acid, and MALDI mass analysis was performed for the analysis of peptides with the α-cyano-4-hydroxycinnamic acid matrix. Then each solution (1.5–2.0 μL) was applied to the MALDI steel plates, air-dried, and used for the MALDI assay. All MALDI spectra were calibrated using standard peptides or protein mixtures II and I (Bremen, Germany, Bruker Daltonic) in the internal or external calibration mode. All MWs (Da) of different peptides are given as the mean ± standard deviation (0.5–1 Da) from 7–10 to three independent MALDI mass specters (Supplementary Data Tables S1 and S2).

## 5. Conclusions

For the first time, we have identified several very large (1.25–1.69 megadaltons) and highly stable multiprotein–peptide complexes in various organs (upper body wall, coelomic fluid, gonads, respiratory trees, and gut) of the sea cucumber Eupentacta fraudatrix. Complexes from different organs contain numerous proteins with MWs >10 kDa; the number and type of these proteins vary considerably among different organs. Furthermore, the complex isolated from the whole organism of E. fraudatrix contains 469 oligopeptides, whereas only 370 peptides were detected in complexes from the five individual organs analyzed. The number of various peptides with MWs <10 kDa in individual organ complexes decreases in the following order: body wall (134) > gut (115) > coelomic fluid (95) > respiratory trees (88) > gonads (70).

In contrast to individual proteins and peptides, multiprotein complexes are capable of interacting with various molecules and cells. Consequently, they can perform expanded functions due to the many molecules located on their surfaces. In cells and biological fluids of various organisms, there are components specific to each organ and biological fluid. These specific components of each complex may be important for carrying out the specialized functions of each individual organ.

## Figures and Tables

**Table 1:** Molecular masses (MWs) of major proteins of the complexes from different organs of *Eupentacta fraudatrix* before and after treatment with DTT, determined from SDS-PAGE analysis data (kDa) *.

Coelomic Fluid		Body Wall		Gut		Gonads		Respiratory Trees	
MWs (kDa)									
Before DTT	After DTT	Before DTT	After DTT	Before DTT	After DTT	Before DTT	After DTT	Before DTT	After DTT
Number of Proteins									
3	6	12	10	3	7	7	5	7	9
280 ± 14.0 *	-	280 ± 14.0 **	-	-	-	280 ± 16.7	-	280 ± 14.1	-
-	-	240 ± 16.8	-	240 ± 15.5	-	-	-	240 ± 15.9	-
-	-	-	-	-	-	215 ± 15.0	-	-	-
-	-	-		-	-	-	122 ± 6.1	-	-
-	-	190 ± 9.5	-	-	-	-	-	-	-
-	-	-	-	-	-	-	-	172 ± 8.6	-
-	-	150 ± 12.1	-	-	-	150 ± 13.8	158 ± 7.2	150 ± 12.5	-
-	140 ± 7.4	-	-	-	-	-	-	-	-
-	-	135 ± 6.7	131 ± 6.5	-	-	-	-	-	-
-	-	125 ± 7.5	120 ± 8.4	-	-	-	-	-	125 ± 8.8
-		-	-	-	-	115 ± 6.9	-		-
-	-	110 ± 6.1	-	-	-	105 ± 5.8	-	-	110 ± 5.5
-	-	70 ± 2.8	-	-	-	-	-	-	
-	-	-	68 ± 5.4	-	69 ± 6.2	65 ± 2.6	65 ± 2.6	68 ± 3.1	69 ± 5.7
60 ± 2.9	60 ± 4.1	62 ± 3.1	60 ± 3.6	-	61 ± 5.6	-	-	-	62 ± 3.1
-	-	-	-	-	-	-	-	-	59 ± 3.5
-	55 ± 2.1	-	56 ± 1.8	-	56 ± 3.1	-	-	-	-
-	-	45 ± 2.7	44 ± 4.0	48 ± 3.1	44 ± 1.9	-	46 ± 2.5	45 ± 2.7	45 ± 1.8
-	40 ± 2.9	-	-	-	38 ± 2.1	-	-	-	40 ± 2.2
-	-	-	35 ± 2.2	-	-	-	-	-	35 ± 2.1
-	-	-	29 ± 1.9	-	30 ± 1.5	-	-	-	-
-	-	25 ± 1.6	26 ± 1.2	-	-	-	-	-	-
-	36 ± 2.0	-	-	-	-	-	-	-	-
-	-	15 ± 0.8	16 ± 0.9	15 ± 1.3	15 ± 1.3	15 ± 1.8	15 ± 1.8	15 ± 1.1	16 ± 0.7
10 ± 2.2	10 ± 3.2	-	-	-	-	-	-	-	-

* All molecular weights (kDa) of proteins are given as the mean ± standard deviation from two to three independent electrophoretic runs. ** The molecular weights (kDa) of some proteins from complexes of different organs did not coincide completely, but taking into account the error in determining these values, they were attributed to the same protein.

**Table 2:** Average molecular weights (MWs) values of 10–20 kDa proteins in stable complexes from different organs of sea cucumbers.

No.	Whole Organism	Body Wall	Respiratory Trees	Gut	Coelomic Fluids	Gonads
Number of Proteins *						
	50	20	14	13	16	19
1	16,809.6 ± 2.3	**-**	**-**	**-**	**-**	**+**
2	15,876.5 ± 2.1	**++** **	**++**	**++**	**++**	**++**
3	15,738.8 ± 2.5	**+** **	**-**	**-**	**-**	**+**
4	15,132.2 ± 2.4	**-**	**-**	**-**	**-**	**+**
5	14,765.5 ± 2.2	**+**	**-**	**-**	**-**	**-**
6	14,754.4 ± 2.3	**+**	**-**	**-**	**-**	**-**
7	14,745.1 ± 2.1	**+**	**-**	**-**	**+**	**-**
8	14,740.2 ± 1.9	**-**	**+**	**-**	**-**	**-**
9	14,726.9 ± 2.3	**+**	**-**	**-**	**-**	**-**
10	14,722.3 ± 2.0	**-**	**++**	**-**	**++**	**-**
11	14,714.2 ± 2.1	**+**	**+**	**-**	**++**	**-**
12	14,710.3 ± 2.3	**-**	**-**	**-**	**+**	**+**
13	14,696.6 ± 2.4	**++**	**+**	**++**	**++**	**++**
14	14,686.5 ± 2.1	**+**	**-**	**-**	**++**	**++**
15	14,673.5 ± 2.2	**-**	**-**	**+**	**+**	**-**
16	14,662.3 ± 2.5	**-**	**-**	**-**	**+**	**+**
17	14,653.7 ± 2.0		**+**	**+**	**++**	**+**
18	14,622.7 ± 2.1	**+**	**-**	**-**	**-**	**-**
19	14,615.1 ± 2.2	**+**	**-**	**-**	**-**	**-**
20	14,601.5 ± 2.0	**+**	**-**	**-**	**-**	**-**
21	14,590.5 ± 2.4	**+**	**-**	**-**	**-**	**-**
22	14,532.5 ± 2.3	**++**	**-**	**-**	**-**	**-**
23	12,235.7 ± 2.0	**-**	**-**	**+**	**-**	**-**
24	11,795.2 ± 2.1	**+**	**-**	**-**	**-**	**-**
25	11,783.6 ± 2.3	**+**	**-**	**-**	**-**	**-**
26	11,766.5 ± 2.4	**+**	**-**	**-**	**-**	**-**
27	11,752.3 ± 2.1	**-**	**++**	**-**	**-**	**-**
28	11,543.0 ± 2.0	**-**	**+**	**-**	**-**	**-**
29	11,733.3 ± 2.4	**+**	**-**	**-**	**+**	**+**
30	11,720.4 ± 2.0	**+**	**-**	**+**	**+**	**-**
31	11,711.8 ± 2.1	**-**	**+**	**+**	**+**	**-**
32	11,702.0 ± 2.3	**-**	**-**	**+**	**-**	**-**
33	11,685.5 ± 2.2	**-**	**-**	**+**	**-**	**-**
34	11,540.6 ± 2.1	**-**	**-**	**+**	**+**	**-**
35	11,526.6 ± 2.4	**+**	**++**	**-**	**-**	**++**
36	11,521.9 ± 2.3	**-**	**++**	**+**	**+**	**++**
37	11,516.2 ± 2.0	**-**	**++**	**++**	**+**	**-**
38	11,454.5 ± 2.3	**-**	**-**	**-**	**-**	**++**
39	11,397.9 ± 2.0	**-**	**-**	**-**	**-**	**++**
40	11,371.0 ± 2.0	**-**	**-**	**-**	**-**	**++**
41	11,361.4 ± 2.1	**-**	**+**	**-**	**-**	**-**
42	11,107.7 ± 2.3	**+**	**-**	**-**	**-**	**-**
43	10,899.3 ± 2.4	**-**	**++**	**-**	**-**	**-**
44	10,892.3 ± 2.0	**-**	**-**	**-**	**-**	**+**
45	10,881.2 ± 2.2	**-**	**-**	**-**	**-**	**+**
46	10,877.6 ± 2.3	**++**	**-**	**-**	**-**	**++**
47	10,864.0 ± 2.4	**-**	**-**	**+**	**-**	
48	10,721.6 ± 2.0	**-**	**-**	**-**	**-**	**+**
49	10,763.0 ± 2.1	**++**	**-**	**-**	**-**	
50	10,702.5 ± 2.2	**-**	**-**	**-**	**+**	**+**

* All molecular weights (Da) of proteins are given as the mean ± standard deviation from 7–10 to three independent MALDI mass specters. ** One plus sign (+) indicates detection of a protein in the complex, while two plus signs (++) correspond to major proteins identified in complexes from different organs. Organ-specific proteins that are not present in other complexes are marked in red.

## Data Availability

All data are given in the article.

## Supplementary Materials

The following supporting information can be downloaded at https://www.mdpi.com/article/10.3390/ijms27146288/s1.

## Abbreviations

The following abbreviations are used:

DTT	dithiothreitol
EDTA	ethylenediaminetetraacetic acid
kDa	kilo Daltons
MW	molecular weight
SDS	sodium dodecyl sulfate
SDS-PAGE	sodium dodecyl sulfate–polyacrylamide gel electrophoresis
MALDI-TOF mass spectrometry	matrix-assisted laser desorption/ionization–mass spectrometry
