# Supplementary material for: Stable and Specific Multiprotein Complexes with High Molecular Masses in Different Organs of the Sea Cucumber Eupentacta fraudatrix
Source: Int J Mol Sci. 2026 Jul 15;27(14):6288. doi: 10.3390/ijms27146288 (PMC13411616; doi:10.3390/ijms27146288)
Supplement: Supplementary file 1 [file ijms-27-06288-s001.zip › ijms-4395534-supplementary.pdf]

Supplementary data

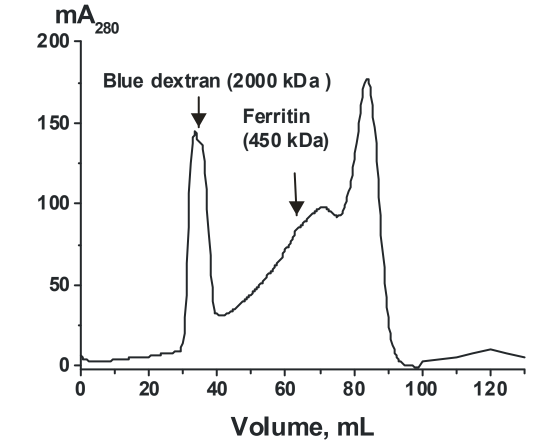

Figure S1. Isolation and analysis of sea cucumber complex. FPLC gel filtration of sea cucumber homogenate proteins on a Sepharose 4B column (A). MW ≈2000 kDa [19].

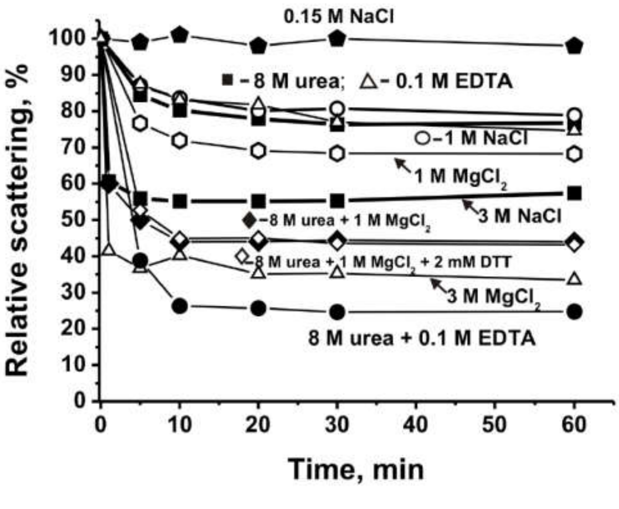

Figure S2. Typical examples of the time changes in the light scattering (LS) of the cucumber complex (0.005 mg/mL) in the presence of 20 mM Tris-HCl buffer (pH 7.5) containing urea, NaCl, $MgCl_2$, DTT, and EDTA in various concentrations and different combinations, detailed in the Figure [19].

Supplementary Table S1 Molecular masses (m/z) of peptides (<10 kDa) of multiprotein complexes of all organs of sea cucumber *Eupentacta fraudatrix*

Numbers of individual peptides and their MWs, Daltons					
1	9650.3	157	6844.6	314	4371.2
2	8733.8	158	6843.1	315	4367.0
3	8724.3	159	6829.7	316	4305.1
4	8615.7	160	6827.1	317	4300.5
5	8609.3	161	6825.0	318	4296.6
6	8598.7	162	6815.1	319	4293.5
7	8595.5	163	6704.5	320	4291.6
8	8593.1	164	6086.2	321	4277.8
9	8579.7	165	6078.3	322	4139.7
10	8577.4	166	6075.9	323	4137.4
11	8570.8	167	6069.1	324	4129.8
12	8564.7	168	6060.0	325	4127.7
13	8559.3	169	6054.2	326	4126.4
14	8281.2	170	6048.7	327	4125.5
15	8262.4	171	6043.4	328	4122.8
16	8260.5	172	6042.4	329	4109.4
17	8252.2	173	6027.7	330	4106.3
18	8534.2	174	6028.7	331	4101.3
19	8530.7	175	6027.2	332	4061.0
20	8245.2	176	6026.3	333	4039.8
21	8232.1	177	6024.9	334	4033.8
22	8221.7	178	6022.8	335	4021.5
23	8219.8	179	6020.8	336	4020.5
24	8217.8	180	6017.3	337	4018.8
25	8215.8	181	5847.4	338	4017.1
26	8214.3	182	5766.9	339	4015.1
27	8211.8	183	5766.9	340	4013.6
28	8207.3	184	5763.8	341	4010.3
29	8202.2	185	5759.7	342	4007.9
30	8201.0	186	5707.7	343	4004.8
31	8196.5	187	5702.1	344	4003.3
32	8194.6	188	5699.2	345	4002.3
33	8158.5	189	5692.3	346	4000.2
34	8155.7	190	5690.3	347	*3998.9*
35	8012.8	191	5688.1	348	3997.1
36	7924.1	192	5685.7	349	3995.8
37	7662.3	193	5684.0	350	3990.8
38	7658.5	194	5682.4	351	3979.4
39	7654.1	195	5615.7	352	3983.6
40	7633.9	196	5610.1	353	3982.4
41	7630.5	197	5604.6	354	3981.7
42	7623.2	198	5602.3	355	3980.2
43	7620.1	199	*5593.8*	356	3979.4
44	7616.6	200	5591.7	357	3977.6
45	7615.0	201	5588.2	358	3970.1
46	7614.3	202	*5586.6*	359	3968.2
47	7613.1	203	5584.7	360	3946.1

48	7607.2	204	5577.2	361	3922.8
49	7611.4	205	5531.5	362	3905.6
50	7610.8	206	5530.1	363	3899.8
51	7608.8	207	5528.7	364	3893.3
52	7607.2	208	5526.3	365	3890.6
53	7602.6	209	5520.3	366	3984.0
54	7593.2	210	5469.9	367	3881.9
55	7521.6	211	5462.4	368	3880.3
56	7545.4	212	5449.6	369	3878.1
57	7540.3	213	5445.1	370	3877.4
58	7537.3	214	5441.4		3874.2
59	7534.1	215	5439.2	371	3870.5
60	7532.7	216	5437.0	372	3869.9
61	7527.0	217	5430.7	373	3865.9
62	7525.9	218	5427.3	374	3864.0
63	7512.2	219	5425.1	375	3850.6
64	7505.9	220	5424.1	376	3848.9
65	7409.9	221	5420.6	377	3847.2
66	7401.7	222	5406.4	378	3846.1
67	7391.2	223	5405.3	379	3845.7
68	7387.7	224	5393.3	380	3843.4
69	7380.7	225	5388.9	381	3840.2
70	7378.9	226	5385.9	382	3837.6
71	7372.8	227	5384.6	283	3833.1
72	*7369.5*	228	5381.5	384	3829.4
73	7367.7	229	5379.4	385	3827.4
74	*7361.5*	230	5377.7	286	3826.3
75	7360.2	231	5374.9	387	3825.5
76	7356.4	232	5373.9	388	3824.4
77	7355.1	233	5370.3	389	3823.7
78	7353.5	234	5318.8	390	3821.9
79	7350.3	235	5312.9	391	3819.0
80	7347.5	236	5309.9	392	3816.4
81	7345.4	237	5304.6	393	3806.0
82	7344.6	238	5302.8	394	3805.1
83	7342.3	239	5301.3	395	3802.3
84	7341.7	240	5302.8	396	3800
85	7340.9	241	5298.0	397	3794.3
86	7339.0	242	5296.5	398	3732.7
87	7336.5	243	5295.7	399	3730.4
88	7334.9	244	5291.4	400	3725.4
89	7331.5	245	5288.7	401	3721.6
90	7327.2	246	5286.5	402	3718.5
91	7321.3	247	5285.3	403	3717.7
92	7294.9	248	5284.5	404	3716.6
93	7275.6	249	5283.0	405	3715.0
94	7272.3	250	5280.7	406	3714.1
95	7271.1	251	5280.9	407	3712.2
96	7269.1	252	5277.0	408	3710.3
97	7267.2	253	5270.3	409	3706.5
98	7161.4	254	5267.6	410	3703.6

99	7140.6	255	5264.5	411	3588.7
100	7122.4	256	*5260.4*	412	3587.0
101	7109.9	257	5259.4	413	3561.7
102	7101.7	258	5257.0	414	3559.2
103	7100.5	259	5256.0	415	3555.2
104	7095.0	260	5254.5	416	3546.0
105	7089.3	261	5252.8	417	3538.5
106	7076.7	262	5246.6	418	3533.8
107	7069.2	263	5236.4	419	3521.3
108	7058.3	264	5235.3	420	3515.7
109	7056.4	265	5234.7	421	3505.0
110	7049.5	266	5232.9	422	3497.8
111	7039.7	267	5232.0	423	3494.9
112	7353.5	268	5229.8	424	3492.5
113	7032.8	269	5225.5	425	3487.9
114	7024.8	270	5224.6	426	3484.1
115	*7022.7*	271	5221.7	427	3483.0
116	7019.5	272	5215.2	428	3482.1
117	7018.3	273	5213.7	429	3481.5
118	7016.1	274	5212.3	430	*3477.2*
119	7014.5	275	5211.9	431	3476.2
120	7011.7	276	5210.1	432	3475.3
121	7009.6	277	5208.4	433	3474.3
122	7008.8	278	5199.1	434	3473.2
123	7007.2	279	5194.3	435	3472.6
124	7004.5	280	5185.0	436	3468.7
125	7002.0	281	5178.8	437	3457.4
126	6997.7	282	5176.8-	438	3453.6
127	6994.7	283	5169.7	439	3451.8
128	6992.7	284	5168.1	440	3444.2
129	*6991.5*	285	5156.3	441	3442.2
130	6990.4	286	5164.1	442	3438.9
131	6983.0	287	5157.4	443	3436.1
132	6981.4	288	5155.7	444	3423.8
133	6979.3	289	5155.0	445	3419.0
139	6977.6	290	5154.2	446	3410.0
134	6975.9	291	5151.7	447	3401.9
135	6973.5	292	5150.6	448	3397.2
136	6972.6	293	5148.6	449	3369.8
137	6970.5	294	5143.5	450	3368.0
138	6966.7	295	5141.4	451	3453.6
139	6955.1	296	5138.4	452	3391.1
140	6954.4	297	5132.1	453	3367.8
141	6953.4	298	5102.5	454	3362.9
142	6951.8	299	5098.4	455	3364.0
143	6950.4	300	5086.2	456	3350.1
144	6949.1	301	5074.5	457	3345.8
245	6948.2	302	5069.3	458	3336.9
146	6944.4	303	5051.4	459	3335.0
147	6930.1	304	5046.8	460	3332.0
148	6928.1	305	5004.4	461	3328.0
149	6925.2	306	4978.7	462	3323.6

150	6917.8	307	4959.7	463	3322.065
151	6908.6	308	4814.4	464	3320.5
152	6901.3	309	4801.8	465	3315.8
153	6887.4	310	4807.8	466	3310.2
154	6855.2	311	4534.6	467	3308.9
155	6853.1	312	4388.3	468	3301.6
156	9650.3	313	4386.7	469	3298.4

*Errors in determined MWs varies from 0.5 to 1.0 Da. In the case of close values, they were considered reliable if they were determined in the same spectrum

Supplementary Table S2. Comparison of peptides of multiprotein complexes from different organs of holothuria *Eupentacta fraudatrix* *

Molecular weights of peptides in Daltons (number of peptide in complex of every individual organ)						
Body shell	Respiratory trees	Gut	Coelomic fluids	Gonads	Peptide number	MW of peptide (Da)
Number of peptides in group						
134	88	115	95	70	№	
-	-	9650.3 (1)	-	-	1	9650.3
-	-	8733.8 (2)	-	-	2	8733.8
-	-	-	-	8724.3 (1)	3	8724.3
8593.1 (1)	-	-	-	-	5	8593.1
8577.4 (2)	-	-	-	-	6	8577.4
-	8570.8 (1)	-	-	-	7	8570.8
8564.7 (3)	-	-	-	-	8	8564.7
8281.2 (4)	-	-	-	-	9	8281.2
8262.4 (5)	-	-	-	-	10	8262.4
8260.5 (6)	-	-	-	-	11	8260.5
-	8252.2 (2)	-	-	-	12	8252.2
-	8232.1 (3)	-	-	-	13	8232.1
-	-	-	8221.7 (1)	-	14	8221.7
82197 (7)	-	8219.7 (3)	-	-	15	8219.8
8217.8 (8)	-	-	-	-	16	8217.8
8215.8 (9)	-	-	8215.8 (2)	-	17	8215.8
8214.3 (10)	-	-	8214.3 (3)	=	18	8214.3
-	8211.8 (4)	8211.8 (4)	-	-	19	8211.8
-	-	8201.0 (5)	-	-	20	8201.0
-	-	-	-	8196.5 (2)	21	8196.5
-	-	-	-	8158.5 (3)	22	8158.5
-	-	-	8012.8 (4)	-	23	8012.8
-	-	7662.3 (6)	-	-	24	7662.3
7658.5 (11)	-	-	-	-	25	7658.5
-	-	7655.1 (7)	-	-	26	
7654.1 (12)	-	-	-	-	27	7654.1
-	7633.9 (5)	7633.9 (8)	-	-	28	7633.9
-	7630.5 (6)	-	-	-	29	7630.5
-	-	-	7623.2 (5)	-	30	7623.2
-	-	-	-	7620.1 (4)	31	7620.1
-	-	-	7616.6 (6)	-	32	7616.6
-	7615.0 (7)	-	-	-	33	7615.0
-	-	7614.1 (9)	-	7614.3 (5)	34	7614.3
-	-	-	7613.1 (7)	-	35	7613.1

-	-	-	-	7612.7 (6)	36	7612.7
7611.4 (13)	-	7611.4 (10)	-	-	37	7611.4
-	7610.8 (8)	7610.8 (11)	7610.8 (8)	-	38	7610.8
7608.8 (14)	-	-	-	-	39	7608.8
-	-	7607.2 (12)	-	-	40	7607.2
-	-	-	7602.6 (9)	-	41	7602.6
7545.4 (15)	-	-		-	43	7545.4
-	-	7540.3 (13)	-	-	43	7540.3
7537.3 (16)	-	-	-	-	44	7537.3
-	-	7534.1 (14)	-	-	45	7534.1
7532.7 (17)	-	-	-	-	46	7532.7
7527.0 (18)	-	-	-	-	47	7527.0
7525.9 (19)	-	-	-	-	48	7525.9
7387.7 (20)	-	-	-	-	49	7387.7
7378.9 (21)	-	-	-	-	50	7378.9
-	-	7372.8 (15)	-	-	51	7372.8
-	-	-	*7369.5* (10)	-	52	*7369.5*
7367.7 (22)	-	-	7367.7 (11)	-	53	7367.7
-	-	-	*7361.5* (12)	-	54	*7361.5*
-	7360.2 (9)	-	-	-	55	7360.2
_	-	-	-	7356.4 (7)	56	7356.4
7355.1 (23)	-	-	-	-	57	7355.1
7353.5 (24)	-	-	-	-	58	7353.5
7350.3 (25)	-	-	7350.3 (13)	-	59	7350.3
-	-	7347.5 (16)	-	7347.5 (8)	60	7347.5
-	-	-	7345.4 (14)	-	61	7345.4
7344.6 (26)	=	-	-	-	62	7344.6
7342.3 (27)	-	-	-	-	63	7342.3
-	-	7341.7 (17)	-	-	64	7341.7
-	-	-	-	7340.9 (9)	65	7340.9
-	-	-	7339.0 (15)	-	66	7339.0
-	-	-	7336.5 (16)	-	67	7336.5
7331.5 (28)	-	-	-	-	68	7331.5
7275.6 (29)	-	-	-	-	69	7275.6
7272.3 (30)	-	-	-	-	70	7272.3
-	-	7271.1 (18)	-	-	71	7271.1
7269.1 (31)	-	-	-	-	72	7269.1
-	-	7267.2 (19)	-	-	73	7267.2
-	-	7161.4 (20)	-	-	74	7161.4
-	-	7140.6 (21)	-	-	75	7140.6
-	-	7122.4 (22)	-	-	76	7122.4
7109.9 (32)	-	-	-	-	77	7109.9
7101.7 (33)	-	7101.7 (23)	-	-	78	7101.7
7100.5 (34)	-	-	-	-	79	7100.5
7095.0 (35)	-	-	-	-	80	7095.0
-	-	7089.3 (24)	-	-	81	7089.3
7076.7 (36)	-	-	-	-	82	7076.7
-	-	7069.2 (25)	-	-	83	7069.2
7058.3 (37)	-	-	-	-	84	7058.3
7056.4 (38)	-	-	-	-	85	7056.4
7049.5 (39)	-	-	-	-	86	7049.5
7039.7 (40)	-	-	-	-	87	7039.7
-	-	-	-	7353.5 (10)	88	7353.5
-	-	-	7024.8 (17)	-	89	7024.8
-	*7022.7* (10)	-	-	-	90	*7022.7*
-	7018.3 (11)	-	-	-	91	7018.3
7016.1 (41)	-	-	-	-	92	7016.1

7014.5 (42)	-	7014.5 (26)	-	-	93	7014.5
-	7011.7 (12)	-	-	-	94	7011.7
-	-	-	7009.6 (18)	-	95	7009.6
-	-	-	-	7008.8 (11)	96	7008.8
-	7007.2 (13)	-	-	-	97	7007.2
-	-	-	7004.5 (19)	-	98	7004.5
7002.0 (43)	-	-	-	-	99	7002.0
-	6997.7 (14)	6997.7 (27)	-	-	100	6997.7
-	-	-	6994.7 (20)	6994.7 (12)	101	6994.7
6992.7 (44)	-	-	-	-	102	6992.7
-	6991.5 (15)	-	-	-	103	*6991.5*
-	-	6990.4 (28)	-	6990.4 (13)	104	6990.4
-	6983.0 (16)	-	-	-	105	6983.0
-	-	-	6981.4 (21)	6981.4 (14)	106	6981.4
6979.3 (45)	-	6979.3 (29)	-	-	107	6979.3
-	-	-	-	6977.6 (15)	108	6977.6
	-	-	6975.9 (22)	-	109	6975.9
-	6973.5 (17)	-	6973.5 (23)	-	110	6973.5
-	-	6972.6 (30)	-	-	111	6972.6
-	6970.5 (18)	-	6970.5 (24)	-	112	6970.5
-	6955.1 (19)	-	-	-	113	6955.1
-	-	6954.4 (31)	-	6954.4 (16)	114	6954.4
6953.4 (46)**	6953.4 (20)	6953.4 (32)	6953.4 (25)	6953.4 (17)	115	6953.4
-	6951.8 (21)	-	-	6951.8 (18)	116	6951.8
-	-	6950.4 (33)	-	-	117	6950.4
-	-	-	-	6949.1 (19)	118	6949.1
-	-	6948.2 (33)	-	-	119	6948.2
-	-	-	-	6930.1 (20)	120	6930.1
-	6928.1 (22)	-	-	-	121	6928.1
6925.2 (47)	-	-	-	-	122	6925.2
6917.8 (48)	-	-	-	-	123	6917.8
6908.6 (49)	-	-	-	-	124	6908.6
-	-	6887.4 (34)	-	-	125	6887.4
6855.2 (50)	-	-	-	-	126	6855.2
6853.1 (51)	-	-	-	-	127	6853.1
-	-	6844.6 (35)	-	-	128	6844.6
6843.1 (52)	-	-	-	-	129	6843.1
6829.7 (53)	-	-	-	-	130	6829.7
6827.1 (54)	-	6827.1 (36)	-	-	131	6827.1
6825.0 (55)	-	-	-	-	132	6825.0
-	6704.5 (23)	-	-	-	133	6704.5
6086.2 (56)	-	-	-	-	134	6086.2
-	-	6078.3 (37)	-	-	135	6078.3
~~6075.9~~ (57)	-	-	-	-	136	~~6075.9~~
-	-	6069.1 (38)	-	-	137	6069.1
6060.0 (58)	-	-	-	-	138	6060.0
-	-	-	-	6054.2 (21)	139	6054.2
6048.7 (59)	-	6048.7 (39)	-	-	140	6048.7
-	-	-	-	6042.4 (22)	141	6042.4
-	-	6027.7 (40)	-	6027.7 (23)	142	6027.7
-	6028.7 (24)	6028.7 (41)	-	-	143	6028.7
-	-	-	6027.2 (26)	-	144	6027.2
6026.3 (60)	-	6026.3 (42)	6026.3 (27)	-	145	6026.3
-	-	-	-	6024.9 (24)	149	6024.9
-	-	6022.8 (43)	-	-	150	6022.8
-	6020.8 (25)	-	-	-	151	6020.8
-	-	-	6017.5 (28)	-	152	5766.9

-	-	-	-	5766.9 (25)	153	5766.9
-	5763.8 (26)	-	-	-	154	5763.8
5759.7 (61)	-	5759.7 (44)	*5759.7* (29)	5759.7 (26)	155	5759.7
5707.7 (62)	-	-	-	-	156	5707.7
5702.1 (63)	-	-	-	-	157	5702.1
-	5699.2 (27)	-	-	-	158	5699.2
-	-	5692.3 (45)	5692.3 (30)	-	159	5692.3
-	5690.3 (28)	-	-	-	160	5690.3
5688.0 (64)	-	5688.1 (46)	5688.2 (31)	-	161	5688.1
5686.4 (65)	5685.7 (29)	-	5685.0 (32)	-	162	5685.7
-	-	5684.0 (47)	-	-	163	5684.0
-	5682.4 (30)	-	*5682.4* (33)	-	164	5682.4
-	-	-	5615.7 (34)	-	165	5615.7
-	5610.1 (31)	-	-	-	166	5610.1
-	-	-	-	5604.6 (27)	167	5604.6
-	5602.3 (32)	-	-	-	168	5602.3
5593.7 (66)	-	-	*5593.8* (35)	-	169	*5593.8*
5591.7 (67)	-	5591.7 (48)	5591.7 (36)	5592.7 (28)	170	5591.7
-	-	-	*5586.6* (37)	-	171	*5586.6*
-	-	5531.5 (49)	-	-	172	5531.5
5530.1 (68)	-	-	-	-	173	5530.1
5528.7 (69)	-	-	-	-	174	5528.7
-	-	5526.3 (50)	-	-	175	5526.3
-	-	5469.9 (51)	-	-	176	5469.9
-	5462.4 (33)	-	-	-	177	5462.4
-	5449.6 (34)	-	-	-	178	5449.6
-	5445.1 (35)	-	-	-	179	5445.1
5441.4 (70)	-	5441.4 (52)	-	-	180	5441.4
-	5439.2 (36)	-	5439.7 (38)	-	181	5439.2
-	-	-	5437.0 (39)	-	182	5437.0
-	-	5430.7 (53)	-	5430.7 (29)	183	5430.7
-	5425.1 (37)	-	-	-	184	5425.1
5421.1 (71)	-	5424.1 (54)	-	-	185	5424.1
-	-	-	-	5406.4 (30)	186	5406.4
-	-	-	5405.3 (40)	-	187	5405.3
-	5393.3 (38)	-	-	-	188	5393.3
-	5385.9 (39)	-	-	-	189	5385.9
-	-	-	5384.6 (41)	5384.6 (31)	190	5384.6
-	-	-	5381.5 (42)	-	191	5381.5
-	5379.4 (40)	5379.4 (55)	5378.4 (43)	-	192	5379.4
-	5377.7 (41)	5377.7 (56)	-	-	193	5377.7
-	-	-	5374.9 (44)	-	194	5374.9
-	-	5373.9 (57)	-	-	195	5373.9
-	5324.9 (42)	-	-	-	196	5324.9
5318.8 (72)	-	-	-	-	197	5318.8
-	5312.9 (43)	-	-	-	198	5312.9
-	-	-	5309.9 (45)	5309.9 (32)	199	5309.9
-	-	5305.6 (58)	5305.6 (46)	5304.6 (33)	200	5304.6
5302.8 (73)	5302.8 (44)	-	-	-	201	5302.8
-	-	5301.3 (59)	5301.3 (47)	5301.3 (34)	202	5301.3
-	5299.2 (45)	-	-	-	203	5302.8
-	-	-	-	5298.0 (35)	204	5298.0
5296.5 (74)	-	5296.5 (60)	5296.5 (48)	-	205	5296.5
-	5295.7 (46)	-	-	-	206	5295.7
-	5291.7 (47)	5291.4 (61)	-	5291.4 (36)	207	5291.4
-	-	-	5288.7 (49)	-	208	5288.7
-	5286.5 (48)	-	5286.5 (50)	-	209	5286.5

-	-	-	-	5285.3 (37)	210	5285.3
5284.5 (75)	-	5284.5 (62)	5284.5 (51)	-	211	5284.5
-	-	-	-	5283.0 (38)	212	5283.0
-	-	-	-	5280.7 (39)	213	5280.7
-	5280.9 (49)	5280..9 (63)	5280.9 (52)	-	214	5280.9
-	5277.0 (50)	5277.0 (64)	*5277.0* (53)	-	215	5277.0
-	-	-	5270.3 (54)	-	216	5270.3
-	-	-	5267.6 (55)	-	217	5267.6
5264.5 (76)	-	-	*5264.5* (56)	-	218	5264.5
-	-	5260.4 (65)	-	-	219	*5260.4*
-	-	-	5259.4 (57)	-	220	5259.4
5257.0 (77)	-	-	-	-	221	5257.0
-	-	-	-	5256.0 (40)	222	5256.0
5254.5 (78)	-	-	-	-	223	5254.5
-	-	-	5252.8 (58)	-	224	5252.8
5246.6 (79)	-	-	-	-	225	5246.6
-	5236.4 (51)	-	5236.4 (59)	-	226	5236.4
-	-	5235.3 (66)	5235.3 (60)	-	227	5235.3
5234.7 (80)	-	-	-	5234.7 (41)	228	5234.7
5232.9 (81)	5232.9 (52)	-	5232.9 (61)	-	229	5232.9
-	-	5232.0 (67)	-	-	230	5232.0
5229.8 (82)	-	5229.8 (68)	-	-	231	5229.8
-	-	-	5225.5 (62)	-	232	5225.5
-	-	-	-	5213.7 (42)	233	5213.7
5212.3 (83)	-	-	-	-	234	5212.3
-	-	-	5211.9 (63)	-	235	5211.9
-	-	5208.4 (69)	-	-	236	5208.4
-	-	-	5185.0 (64)	-	237	5185.0
5176.8 (84)	-	-	5176.8 (65)	-	238	5176.8-
-	-	5169.7 (70)	-	-	239	5169.7
5168.1 (85)	-	-	-	-	240	5168.1
-	5156.3 (53)	-	-	-	241	5156.3
-	-	-	5164.1 (66)	-	242	5164.1
-	-	-	5157.4 (67)	-	243	5157.4
5155.7 (86)	-	-	-	5155.7 (43)	244	5155.7
-	-	5155.0 (71)	-	-	245	5155.0
-	-	-	5154.2 (68)	-	246	5154.2
5151.7 (87)	5151.7 (54)	5152.7 (72)	5151.7 (69)	5151.7 (44)	247	5151.7
-	-	5150.6 (73)	-	5150.6 (45)	248	5150.6
-	-	-	5141.4 (70)	-	249	5141.4
-	-	-	5138.4 (71)	-	250	5138.4
-	-	5132.1 (74)	-	-	251	5132.1
-	-	5074.5 (75)	-	-	252	5074.5
-	-	5069.3 (76)	-	-	253	5069.3
-	-	5046.8 (77)	-	-	254	5046.8
-	-	-	-	4978.7 (46)	255	4978.7
-	-	4534.6 (78)	-	-	256	4534.6
-	4305.1 (55)	-	-	-	257	4305.1
-	-	4293.5 (79)	-	-	258	4293.5
-	-	-	-	4270.2 (47)	259	4293.5
-	-	4139.7 (80)	-	-		4139.7
-	4137.4 (56)	-	-	-	260	4137.4
4129.8 (88)	4129.8 (57)	4129.8 (81)	4129.8 (72)	4129.8 (48)	261	4129.8
-	-	4127.7 (82)	4127.7 (73)	4127.7 (49)	262	4127.7
-	4126.4 (58)	-	-	-	263	4126.4
4125.5 (89)	-	4125.5 (83)	-	-	264	4125.5
-	-	-	-	4122.8 (50)	265	4122.8

-	-	4106.3 (84)	-	-	266	4106.3
4061.0 (90)	-	-	-	-	267	4061.0
4039.8 (91)	-	-	-	-	268	4039.8
4033.8 (92)	-	-	-	-	269	4033.8
-	4021.5 (59)	4021.5 (85)	-	-	270	4021.5
4020.5 (93)	-	-	4020.5 (74)	-	271	4020.5
4018.8 (94)	-	-	-	-	272	4018.8
4017.1 (95)	-	-	-	-	273	4017.1
-	4015.1 (60)	-	4015.1 (75)	-	274	4015.1
-	-	4013.6 (86)	-	-	275	4013.6
-	4007.9 (61)	4007.9 (87)	-	4007.9 (51)	276	4007.9
-	-	-	4004.8 (76)	4004.8 (52)	277	4004.8
-	4003.3 (62)	4003.3 (88)	-	-	278	4003.3
4002.3 (96)	-	-	4002.3 (77)	-	279	4002.3
4000.2 (97)	-	-	-	-	280	4000.2
-	-	-	*3998.9* (78)	-	281	*3998.9*
-	-	3997.1 (89)	-	3997.1 (53)	282	3997.1
-	-	3995.8 (90)	-	-	283	3995.8
-	3990.8 (63)	-	-	-	284	3990.8
-	3979.4 (64)	-	-	-	285	3979.4
3983.6 (98)	-	3983.6 (91)	3983.6 (79)	3983.6 (54)	286	3983.6
3982.4 (99)	3982.4 (65)	3982.4 (92)	-	-	287	3982.4
-	3981.7 (66)	3981.7 (93)	3981.7 (80)	3981.7 (55)	288	3981.7
-	-	-	-	3980.2 (56)	289	3980.2
3979.4 (100)	-	-	3979.4 (81)	-	290	3979.4
-	-	3977.6 (94)	-	3977.6 (57)	291	3977.6
-	3970.1 (67)	-	-	-	292	3970.1
3946.1 (101)	-	-	-	-	293	3946.1
3922.8 (102)	-	-	-	-	294	3922.8
3905.6 (103)	-	-	-	-	295	3905.6
3899.8 (104)	-	-	-	-	296	3899.8
3893.3 (105)	-	-	-	-	297	3893.3
-	3890.6 (68)	-	-	-	298	3890.6
-	3984.0 (69)	-	-	-	299	3984.0
3881.9 (106)	-	-	-	-	300	3881.9
-	3880.3 (70)	-	3880.3 (82)	-	301	3880.3
3878.1 (107)	-	-	-	-	302	3878.1
3877.4 (108)	-	-	-	-	303	3877.4
-	3874.2 (71)	-	-	-	304	3874.2
-	-	-	-	3870.5 (58)	305	3870.5
3869.9 (109)	-	-	-	-	306	3869.9
3865.9 (110)	*3865.9* (72)	-	-	-	307	3865.9
3864.0 (111)	-	-	-	-	*308*	3864.0
-	-	3850.6 (95)	-	-	*309*	3850.6
3848.9 (112)	-	-	-	-	*310*	3848.9
-	-	3847.2 (96)	-	-	*311*	3847.2
-	3846.1 (73)	-	-	-	312	3846.1
3845.7 (113)	-	-	3845.7 (83)	-	313	3845.7
3843.4 (114)	3843.4 (74)	-	-	-	314	3843.4
-	-	3840.2 (97)	-	-	315	3840.2
-	3837.6 (75)	3837.6 (98)	*3837.6* (84)	-	316	3837.6
-	3833.1 (76)	-	-	-	317	3833.1
3829.4 (115)	-	3829.4 (99)	-	-	318	3829.4
-	3827.4 (77)	-	3827.4 (85)	-	*319*	3827.4
-	-	3826.3 (100)	3826.3 (86)	3826.3 (59)	320	3826.3
3825.5 (116)	-	3825.5 (101)	3825.5 (87)	3825.5 (60)	321	3825.5
-	3824.4 (78)	3824.4 (102)	3824.4 (88)	-	322	3824.4

3823.7 (117)	-	-	-	-	323	3823.7
-	3821.9 (79)	-	3821.9 (89)	-	324	3821.9
-	-	-	-	3819.0 (61)	325	3819.0
3816.4 (118)	3816.4 (80)	-	-	-	326	3816.4
3806.0 (119)	-	-	-	-	327	3806.0
-	-	-	3805.1 (90)	-	328	3805.1
-	3802.3 (81)	3802.3 (103)	-	-	329	3802.3
3732.7 (120)	-	-	-	-	330	3732.7
-	3730.4 (82)	-	-	-	331	3730.4
-	3725.4 (83)	-	-	-	332	3725.4
-	-	-	-	3721.6 (62)	333	3721.6
-	-	3718.6 (104)	3718.6 (91)	3718.5 (63)	334	3718.5
3717.7 (121)	-	-	-	-	335	3717.7
-	3716.6 (84)	-	-	-	336	3716.6
3715.0 (122)	-	-	-	3715.0 (64)	337	3715.0
-	-	3714.1 (105)	-	-	338	3714.1
-	3710.3 (85)	-	-	-	339	3710.3
-	-	3703.6 (106)	-	-	340	3703.6
3588.7 (123)	-	-	-	-	341	3588.7
-	-	-	-	3587.0 (65)	342	3587.0
3561.7 (124)	-	-	-	-	343	3561.7
3559.2 (125)	-	-	-	-	344	3559.2
3555.2 (126)	-	-	-	-	345	3555.2
-	-	-	-	3546.0 (66)	346	3546.0
3538.5 (127)	-	-	-	-	347	3538.5
-	-	-	-	3533.8 (67)	348	3533.8
3521.3 (128)	-	-	-	-	349	3521.3
3505.0 (129)	-	-	-	-	350	3505.0
3497.8 (130)	-	-	-	-	351	3497.8
-	-	3494.9 (107)	-	-	352	3494.9
3492.5 (131)	-	-	-	-	353	3492.5
-	-	3487.9 (108)	-	-	354	3487.9
3484.6 (132)	-	-	-	-	355	3487.9
-	3483.0 (86)	-	-	-	356	3483.0
-	-	3482.1 (109)	-	-	357	3482.1
-	-	-	*3477.2* (92)	-	358	*3477.2*
-	-	3476.2 (110)	-	-	359	3476.2
-	-	-	-	3475.3 (68)	360	3475.3
3474.3 (133)	3474.3 (87)	3474.3 (111)	3474.3 (93)	3474.3 (69)	361	3474.3
-	-	-	3473.2 (94)	-	362	3473.2
-	-	3472.6 (112)	-	-	363	3472.6
-	-	-	3468.7 (95)	-	364	3468.7
-	-	3444.2 (113)	-	-	365	3444.2
-	-	3442.2 (114)	-	-	366	3442.2
-	3371.3 (88)	-	-	-	367	3371.3
-	-	3368.0 (115)	-	-	368	3368.0
-	-	-	-	3336.9 (70)	369	3336.9
3335.0 (134)	-	-	-	-	370	3335.0

*Table S2 contains only those very close MWs, which corresponded to closely spaced peaks in the same spectrum.

** Peptides revealed in 5 or 4 complexes are marked with red.
